# Supplementary material for: Accelerating inference in genomic and proteomic foundation models via speculative decoding
Source: Bioinformatics. 2026 Jul 31;42(8):btag579. doi: 10.1093/bioinformatics/btag579 (PMC13474806; doi:10.1093/bioinformatics/btag579)
Supplement: Supplementary file 1 [file btag579_supplementary_data.docx]

**Supplementary Material**

**Supplementary Figure 1. Effect of layer selection strategy on speculative decoding performance.** Acceptance rate (α) as a function of draft depth for three layer selection strategies: prefix truncation (first-N layers), suffix truncation (last-N layers), and mixed/non-contiguous layer selection. Results are shown for ProtGPT2 and ProGen2. Prefix truncation consistently achieves higher acceptance rates across all draft depths, indicating better agreement between draft and target models.

**Supplementary Figure 2: Mean acceptance rate for ProGen2 speculative decoding across draft architectures.** Mean draft-token acceptance rate as a function of speculation window size *L* for ProGen2, comparing a pretrained ProGen2-small draft with truncated drafts derived from the ProGen2-xlarge target using the first 2–6 Transformer layers. Lines show the mean over runs and shaded regions indicate variability across configurations.

**Supplementary Figure 3: Throughput of ProGen2 speculative decoding for different models.** Mean decoding throughput (tokens per second) for ProGen2 when using truncated drafts with 2–6 Transformer layers, a pretrained ProGen2-small draft, and the KV-cached ProGen2-xlarge target-only baseline. Bars show mean tokens/s over repeated runs and error bars denote variability across runs.

**Supplementary Figure 4. Higher-order sequence statistics under speculative decoding.** A. DNAGPT 6-mer frequency spectrum comparison between baseline and speculative decoding outputs; each point represents a hexanucleotide frequency (Pearson r and Jensen–Shannon divergence shown). B. Per-sequence Shannon entropy distributions for DNAGPT, ProGen2, and ProtGPT2 generations. C. Protein dipeptide frequency comparison for ProGen2 and ProtGPT2 outputs. Across all panels, higher-order sequence statistics remain consistent between decoding strategies.

**Supplementary Tables**

**Supplementary Table 1: Sequences / Prompts used for the experiments on the three models.**

[**Sequences / Prompts**](https://docs.google.com/spreadsheets/d/13zxMYsq6i78Cwz-GPydMfqnJ_m6MA2upPp5OYjI6e0k/edit?usp=sharing)

**Supplementary Table 2: Model parameters per truncated layer count**

[**Model parameters**](https://docs.google.com/spreadsheets/d/1sk4BqXekcdMxYRy_uyOgyTvOVObFPWisSykudr84kx0/edit?usp=sharing)

**Supplementary Table 3: Analytical metrics captured for the experiments of the three models.**

[**Analytical metrics**](https://docs.google.com/spreadsheets/d/10otewtorSYnm60kqRQyl9UtKiMBKAxlA5xRX3inN3eM/edit?usp=sharing)
